# Spider mite males undress females to secure the first mating

**DOI:** 10.1016/j.isci.2023.107112

**Published:** 2023-07-07

**Authors:** Peter Schausberger, Thi Hanh Nguyen, Mustafa Altintas

**Affiliations:** 1 Department of Behavioral and Cognitive Biology, University of Vienna, Djerassiplatz 1, 1030 Vienna, Austria

**Keywords:** Entomology, Evolutionary biology, Zoology

## Abstract

Intense mate competition favors the evolution of extraordinary mating strategies such as the ability of males to identify premature females that are close to becoming mature and associate with them until they are sexually receptive (dubbed precopulatory mate guarding). Owing to possible take-overs by rival males, precopulatory guarding is a high-risk, time- and energy-intensive strategy. Here, we provide experimental evidence that guarding spider mite males undress females by actively removing the exuvia during ecdysis to adult; females undressed by guarding males eclose faster than unguarded females. Such behavior is adaptive because it maximizes the guard's chances of being the first mate.

## Highlights

• Males of two-spotted spider mites guard premature females
• Precopulatory guarding evolved because of first male sperm precedence
• Guarding spider mite males strip off the exuvia of premature females
• Undressing the female maximizes the guard’s chances to being the first mate

## Graphical abstract

**Figure:**
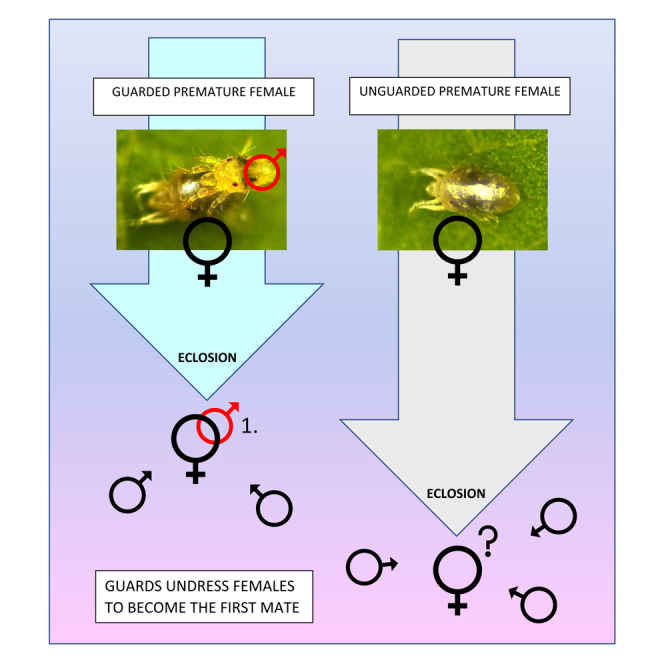

## Introduction

Precopulatory guarding behavior evolves from intense intrasexual competition and occurs primarily in species with short time windows for fertilization during the female reproductive cycle, monandry (one mating partner in life or per breeding season) and/or first male sperm precedence.^1^^,^^2^^,^^3^^,^^4^ It is common, among others, in crustaceans,^5^ butterflies,^6^ and mites.^7^ Precopulatory guarding is a risky “all or nothing” investment because it does not necessarily lead to fertilization of the guarded female. In many species, take-overs by rival males and/or interference and displacement of guards during, or shortly after, female emergence may occur.^3^ Displaced guards incur enormous costs because they expended time and energy in a prospective mate without obtaining any reward, that is, securing copulation. Other costs may arise because guarding is traded off against search for other receptive females and foraging (no energy intake), and/or entails higher vulnerability to predators.^8^ Given these exceptional costs, mate guarding theories predict that males should evolve strategies that allow shortening the guarding duration and maximizing the chances of indeed fertilizing the guarded female.^1^^,^^3^^,^^4^ This may, for example, be achieved by flexibly adjusting the start and duration of guarding to female age, the operational sex ratio and level of male competition. Male guarding propensity should decrease with increasing female-biased sex ratio and increase with increasing male-biased sex ratio.^3^ At high density and under intense competition, males should guard more likely and earlier before female molt. When take-overs and displacement are possible, the guarding costs accumulate over time and culminate at female emergence.^3^ In such species, guards are under strong selective pressure to evolve behaviors preventing take-overs and displacement by rival males shortly before, during, and after female emergence. Male butterflies, for example, can practice pupal mating, that is, they inseminate the female while she is still covered by the pupal case.^6^ Another strategy could be accelerating ecdysis by stimulating the female and/or actively removing her exuvia, i.e. undressing the female, to more quickly get access to her genital opening. Here we report one such exceptional strategy in plant-inhabiting two-spotted spider mites *Tetranychus urticae*, where males guard premature females before copulation. Copulation occurs by the male slipping beneath the female from behind, bending the tip of his abdomen upwards and inserting his aedeagus into the ventrally located genital opening of the female. Pre-copulatory guarding evolved because fertilization of spider mite females is reproductively most advantageous immediately after molting to adult, though females may mate more than once, and first male sperm precedence.^9^^,^^10^ First male mates sire all daughters (sons are haploid and only receive maternal genes) unless another mating occurs shortly after the first mating.^10^ Female deutonymphs become quiescent before molting to adult; the quiescent stage is called teleiochrysalis and lasts about 24 h, with female teleiochrysalis developing more slowly in male absence.^11^ In the final phase before ecdysis, teleiochrysalis females assume a silvery appearance, which lasts about 2 h. This is a highly critical phase for the guarding males. Silvery teleiochrysalis females and just emerged, sexually receptive females are especially attractive to non-guarding rival males, because of releasing more or more attractive pheromones.^7^

## Results and discussion

**Figure 1:**
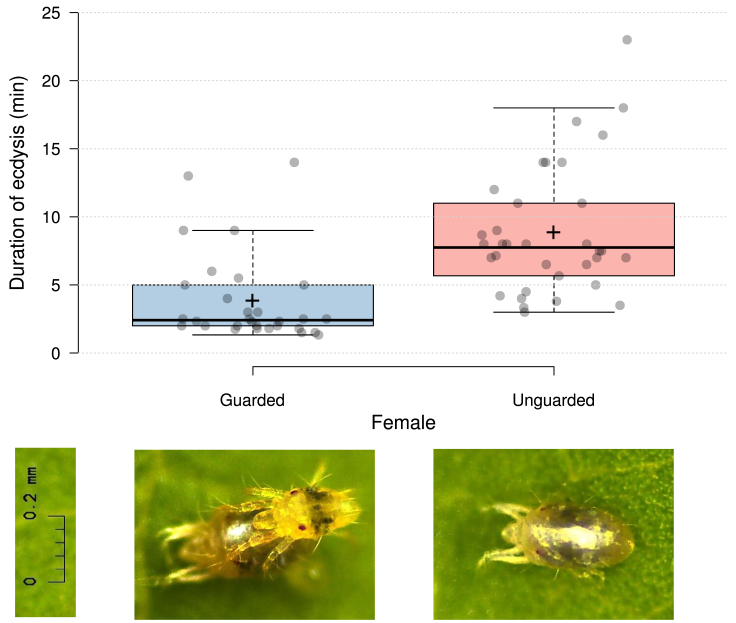
Duration of ecdysis, from cracking of the exuvia to being accessible to males for mating, of guarded and unguarded two-spotted spider mite females, *Tetranychus urticae* Boxes indicate the 25^th^ and 75^th^ percentiles; whiskers extend 1.5 times the interquartile range from the 25^th^ and 75^th^ percentiles; solid horizontal lines inside boxes show the medians, crosses are the sample means; closed circles indicate individual data (n = 30 and 34 for guarded and unguarded females; GLM, p < 0.001 between guarded and unguarded teleiochrysalis females). See also Videos S1, S2, and S3.

We experimentally assessed behavioral strategies of guarding males to secure the first mating and compared ecdysis of guarded and unguarded females. Guarded females eclosed significantly faster than unguarded females (GLM; Wald _X_^2^ = 24.291, p < 0.001; Figure 1). Before cracking of the exuvia, males were observed drumming the female dorsum with their forelegs, which may function to stimulate the female to bulge and start cracking the exuvia (Videos S1 and S2). Upon cracking of the exuvia (indicated by a cross-line appearing in the middle of the dorsum), using their pedipalps (limb-like mouthparts adjacent to the chelicerae), males pulled off the posterior half of the exuvia to expose the ventrally located genital opening of the female (Videos S1 and S2). This behavior was performed by every guarding male observed during the experiment. As a result, guarded females shed the exuvia faster, and exposed their genital opening earlier than did unguarded females (Figure 1). Guarded females usually (all but one of observed cases) first shed, with the help of the male, the posterior part of the exuvia, while unguarded females usually (all observed cases) first pulled out from the anterior part (Videos S1, S2, and S3). Mating took immediately place after the males had uncovered the posterior part of the female idiosoma, where the genital opening is located ventrally. Some males inseminated the female while the anterior part of the female’s body was still partially covered by the exuvia. We observed one freshly molted female being reluctant to allow access to her genital opening (by pressing the posterior ventral part of her body to the leaf surface and by this way preventing the male from slipping beneath her body) to a male that had been little active in assisting the female to get rid of her skin. Eagerness in undressing may indirectly benefit females if this behavior is indicative of male quality and females adjust their accessibility accordingly. Our study provides the first stringent experimental evidence that guarding arthropod males undress females during ecdysis to make them more rapidly accessible for copulation. Such behavior is adaptive because it maximizes the chances to be the first mate and secures siring the offspring.

Video S1. Undressing of the female by the guarding male, related to Figure 1

Video S2. Close-up of undressing of the female by the guarding male, related to Figure 1

Video S3. Ecdysis of an unguarded female, related to Figure 1

### Limitations of the study

Our study documents an exceptional behavior displayed by guarding spider mite males in the final phase of ecdysis of the guarded female. Guards strip off the exuvia of the females to make them earlier accessible for mating. Documentation and quantification of such behavior requires strict standardization of the experimental design, which was achieved by observing single females guarded by one male in absence of rival males. Although guards show the undressing behavior also in presence of rival males (personal observations), and close proximity to the emerging female is decisive for becoming the first mate,^7^^,^^12^^,^^13^ the success rate of the undressing behavior in securing the first mating under the risk of displacement or take-overs by non-guarding rival males remains to be tested. Another open question is whether sneaker and fighter males,^12^^,^^13^ which both guard premature females, differ in undressing behavior.

## STAR★Methods

### Key resources table

**Table:** 

REAGENT or RESOURCE	SOURCE	IDENTIFIER
**Deposited data**		

Raw data	This paper	figshare: https://doi.org/10.6084/m9.figshare.22596940

**Experimental models: Organisms/strains**		

*Tetranychus urticae* (green form)	Collected on bean plants in a greenhouse in Vienna, Austria (this paper)	N/A
*Phaseolus vulgaris* var. Maxi BIO	Austrosaat	https://www.austrosaat.at/

**Software and algorithms**		

IBM SPSS version 28.0	IBM, Armonk, NY, USA	N/A
BoxPlotR	Spitzer et al. (2014)^14^	http://shiny.chemgrid.org/boxplotr/

**Other**		

Leica DMS1000	Leica Microsystems	https://www.leica-microsystems.com/
Leica M80	Leica Microsystems	https://www.leica-microsystems.com/

### Resource availability

#### Lead contact

Further information and requests for resources and reagents should be directed to and will be fulfilled by the lead contact, Peter Schausberger (peter.schausberger@univie.ac.at).

#### Material availability

This study did not generate new unique reagents.

### Experimental model and subject details

#### Origin and rearing of experimental animals

Two-spotted spider mites *Tetranychus urticae* (green form) used in the experiment came from a population founded by specimens collected on common bean plants *Phaseolus vulgaris* in a greenhouse in Vienna. In the laboratory the mites were reared on whole bean plants *P. vulgaris* var. Maxi at 23 ± 1°C, 60 ± 5% RH, 16:8 L:D.

### Method details

#### Experimental procedure

Teleiochrysalis females, that is, female deutonymphs in the quiescent phase, were randomly withdrawn from the lab-reared population and singly placed on leaf discs (1 cm Ø), punched out from detached bean leaves using a cork borer, placed adaxial side up on moist filter paper resting on cotton pads inside acrylic cages.^12^ Each acrylic cage consisted of a closed Petri dish (5 cm Ø, height 1.5 cm) with a mesh-covered (mesh size 0.05 mm) ventilation opening (1.3 cm Ø) in the lid (SPL Life Sciences Co., Ltd., S-Korea). Four males randomly withdrawn from the rearing were added to the teleiochrysalis female (for the guarded female treatment) or the teleiochrysalis female was left without males (for the unguarded female treatment). The experiment was conducted in a fully climatized laboratory at 23 ± 1°C, 60 ± 2% RH and natural daylight. Cages with males were checked in irregular intervals (15–30 min) for occurrence of the first guarding male. As soon as the first guard appeared, the other three males were removed from the disc. After that, cages with both guarded and unguarded females were monitored in irregular intervals (45 min–90 min) for teleiochrysalis females entering the silvery phase. Between observations, cages were stored in an environmental chamber (Panasonic MLR-352H) at 23 ± 1°C, 60 ± 1% RH and 16:8 h L:D. Teleiochrysalis females appear silvery from about 2 h before ecdysis until ecdysis. They appear silvery because the exuvia separates from the new skin and air fills the gap between the old and new skin. Upon entering the silvery phase, both guarded and unguarded females were observed continuously, using either an analog stereomicroscope (Leica M80) or on-screen using a digital microscope (Leica DMS1000). About half of the females were videotaped during ecdysis. Ecdysis of females starts with cracking of the exuvia, which is indicated by a cross-line appearing in the middle of the dorsal side. The duration of ecdysis was defined as the time elapsed from cracking of the exuvia to females exuviating, i.e. shedding the exuvia. For guarded females this was then also the time of their genital opening being exposed and becoming accessible to insemination by the guarding male.

### Quantification and statistical analysis

Ecdysis duration was compared between guarded and unguarded females by a generalized linear model (GLM; normal distribution, identity link) using IBM SPSS version 28 (IBM, Armonk, NY, USA). A digital microscope (Leica DMS1000) was used to take the photographs presented in Figure 1 and the videos presented as supplemental files. The boxplots in Figure 1 were created using BoxPlotR.^14^

## Data Availability

The raw data underlying this study have been deposited at figshare and are publicly available as of the date of publication. DOI is listed in the key resources table.
